# Technical assessment of a mobile CT scanner for image‐guided brachytherapy

**DOI:** 10.1002/acm2.12738

**Published:** 2019-10-02

**Authors:** Nicole E. Chernavsky, Marc Morcos, Pengwei Wu, Akila N. Viswanathan, Jeffrey H. Siewerdsen

**Affiliations:** ^1^ Department of Biomedical Engineering Johns Hopkins University Baltimore MD USA; ^2^ Department of Radiation Oncology and Molecular Radiation Sciences Johns Hopkins University Baltimore MD USA

**Keywords:** brachytherapy, dose, image guidance, image quality, mobile CT

## Abstract

**Purpose:**

The imaging performance and dose of a mobile CT scanner (Brainlab Airo®, Munich, Germany) is evaluated, with particular consideration to assessment of technique protocols for image‐guided brachytherapy.

**Method:**

Dose measurements were performed using a 100‐mm‐length pencil chamber at the center and periphery of 16‐ and 32‐cm‐diameter CTDI phantoms. Hounsfield unit (HU) accuracy and linearity were assessed using materials of specified electron density (Gammex RMI, Madison, WI), and image uniformity, noise, and noise‐power spectrum (NPS) were evaluated in a 20‐cm‐diameter water phantom as well as an American College of Radiology (ACR) CT accreditation phantom (Model 464, Sun Nuclear, Melbourne, FL). Spatial resolution (modulation transfer function, MTF) was assessed with an edge‐spread phantom and visually assessed with respect to line‐pair patterns in the ACR phantom and in structures of interest in anthropomorphic phantoms. Images were also obtained on a diagnostic CT scanner (Big Bore CT simulator, Philips, Amsterdam, Netherlands) for qualitative and quantitative comparison. The manufacturer’s metal artifact reduction (MAR) algorithm was assessed in an anthropomorphic body phantom containing surgical instrumentation. Performance in application to brachytherapy was assessed with a set of anthropomorphic brachytherapy phantoms — for example, a vaginal cylinder and interstitial ring and tandem.

**Result:**

Nominal dose for helical and axial modes, respectively, was 56.4 and 78.9 mGy for the head protocol and 17.8 and 24.9 mGy for the body protocol. A high degree of HU accuracy and linearity was observed for both axial and helical scan modes. Image nonuniformity (e.g., cupping artifact) in the transverse (*x*,*y*) plane was less than 5 HU, but stitching artifacts (~5 HU) in the longitudinal (*z*) direction were observed in axial scan mode. Helical and axial modes demonstrated comparable spatial resolution of ~5 lp/cm, with the MTF reduced to 10% at ~0.38 mm^−1^. Contrast‐to‐noise ratio was suitable to soft‐tissue visualization (e.g., fat and muscle), but windmill artifacts were observed in helical mode in relation to high‐frequency bone and metal. The MAR algorithm provided modest improvement to image quality. Overall, image quality appeared suitable to relevant clinical tasks in intracavitary and interstitial (e.g., gynecological) brachytherapy, including visualization of soft‐tissue structures in proximity to the applicators.

**Conclusion:**

The technical assessment highlighted key characteristics of dose and imaging performance pertinent to incorporation of the mobile CT scanner in clinical procedures, helping to inform clinical deployment and technique protocol selection in brachytherapy. For this and other possible applications, the work helps to identify protocols that could reduce radiation dose and/or improve image quality. The work also identified areas for future improvement, including reduction of stitching, windmill, and metal artifacts.

## 1 INTRODUCTION

Mobile systems for intraoperative 3D imaging have become prevalent over the last decade. One such system is the Brainlab Airo (Brainlab Airo®, Munich, Germany), a mobile CT scanner based on a 32‐row detector with a large bore size (107‐cm inner diameter), small footprint, and slim gantry design. Potentially advantageous features include improved image quality compared to cone‐beam CT (CBCT) and reduced cost / increased flexibility compared to a conventional diagnostic multidetector CT (MDCT) scanner in a dedicated simulation room. As such systems are introduced, rigorous technical assessment can help guide clinical implementation and development of future applications in image‐guided interventions.

Previous studies reported the performance of the mobile CT scanner within the context of its primary indication in image‐guided spine surgery. Weir et al.^1^ assessed the Airo in terms of its imaging performance in comparison to MDCT for image‐guided surgery, including dosimetric characterization and image quality analysis. Their work showed spatial resolution up to 4 lp/cm in the head field‐of‐view (FOV) for the mobile scanner, compared to 7 lp/cm resolution from a Siemens Sensation 64‐slice MDCT scanner for the same FOV.^1^ The Airo was also found to exhibit higher radiation dose than the Sensation 64 for comparable technique factors (50% increase for head, 85% increase for body phantom), and ring‐like artifacts were noted as contributors to increased low‐frequency noise in the image NPS.^1^

The system was further evaluated for application in spine surgery by Hecht et al.,^2^ who assessed the accuracy and workflow for navigated spinal instrumentation, reporting a screw placement accuracy rate of 95.9%.^3^ Similarly, Scarone et al.^3^ performed a retrospective study comparing the Airo to the O‐arm (Medtronic, Dublin, Republic of Ireland) for transpedicular screw fixation in thoracic and lumbar spine surgery. For comparable scan protocols, a decrease in mean radiation exposure was reported for the Airo compared to the O‐arm (15.8 vs 19.1 mSv, respectively), and mean operating time was found to be similar for the two systems. The authors observed a possible reduction in the rate of screw repositioning (1.4% for cases conducted with the Airo compared to 4.3% for the O‐arm), but the overall accuracy and rate of screw malplacement were comparable for the two systems.^3^

For application in image‐guided proton therapy, the study reported by Oliver et al.^4^ included comparison of mobile CT performance to a CBCT system and two MDCT systems. The limiting spatial resolution was reported at 2.1 lp/cm for the Airo, compared to 4.0 lp/cm for the Brilliance Big Bore CT simulator (Philips, Amsterdam, Netherlands) and 3.7 lp/cm for the EDGE CBCT system (Varian, Palo Alto, CA). Compared to the Philips CT simulator, the Airo was found to exhibit a 60% higher dose for head protocols and 8% higher dose for abdomen protocols. Despite the lower spatial resolution, localization accuracy was within 0.6° and 0.5 mm, which was concluded to be sufficient for therapy guidance.^4^

The work reported below is distinct from previous publications in several important respects. A technical assessment guiding the selection of technique protocols for the Airo has yet to be described. Specifically, the results shown below provide a thorough evaluation of imaging performance for both helical and axial modes, and the effect of various technical performance characteristics on image quality in a range of pertinent anatomical sites is evaluated. Accordingly, the work identifies distinct sources of helical and axial mode image artifacts that may be significant for some imaging tasks — viz., helical mode windmill sampling artifacts (especially pronounced about high‐contrast, high‐frequency structures, such as bones or metal instrumentation) and axial mode stitching artifacts that present nonuniformity in the longitudinal direction. We also investigate the effect of centering errors (i.e., patient misaligned from isocenter) on image uniformity, owing to the effect of such errors on bowtie filter calibration. The performance of the manufacturer’s metal artifact reduction (MAR) algorithm is investigated, and emphasis throughout is primarily on soft‐tissue visualization tasks pertinent to soft‐tissue interventions (e.g., liver lesions, prostate, and cervix). Finally, we focus the studies upon the growing scope of clinical application of this mobile CT scanner in brachytherapy — a context within which its performance characteristics have yet to be evaluated and interpreted with respect to relevant imaging tasks.

Brachytherapy is an important modality for both definitive and adjuvant treatment of cervical, endometrial, and prostate cancers,^5^, ^6^, ^7^ and significant developments over the last two decades have increased the use of 3D image guidance in brachytherapy.^8^ Current evidence suggests that 3D image‐guided brachytherapy improves local control compared with conventional brachytherapy;^9^ however, the integration of imaging and treatment delivery in brachytherapy is often limited by logistical and space constraints. Patient transfer between the imaging and treatment areas is often the only viable solution and increases the potential for motion of the applicator with respect to target and normal tissues and creates uncertainty in treatment delivery.^10^ Uncertainty can be partially mitigated by using intraoperative ultrasound to assist with placement of the brachytherapy applicator or by implementing fixation mechanisms to restrict external patient movement. Limitations of the ultrasound approach include image quality / interpretation, image artifacts arising from the brachytherapy applicator, and challenges in modifying the treatment plan.^11^ Limitations of the fixation mechanisms include the inability to prevent internal organ deformation (e.g., bowel, bladder) between imaging and dose delivery.

The incorporation of a CT system in the treatment room would enable the integration of applicator insertion, imaging, and treatment delivery into a single location without the need to transfer the patient. Additionally, the improved workflow efficiency that an integrated treatment room provides could improve patient safety for procedures involving anesthesia.^12^ Previous reports have described integrated image‐guided brachytherapy suites. For example, the Advanced Multimodality Image Guided Operating (AMIGO) suite integrates CT, positron emission tomography (PET), magnetic resonance imaging (MRI), and ultrasound.^12^ While the AMIGO suite uses an impressive array of imaging modalities at the time of implantation, patients must then be transferred to radiation oncology where there is adequate shielding for brachytherapy treatment. An integrated CBCT brachytherapy suite is described at the MAASTRO clinic in the Netherlands;^13^ however, the quality of CBCT is known to be inferior to MDCT, challenged in particular with respect to soft‐tissue visualization. In this work, we describe the use of a mobile CT unit in a shielded brachytherapy suite, informed by a technical assessment of the CT scanner.

The technical assessment reported below examines the imaging performance and radiation dose for the Airo mobile CT scanner, including a variety of manufacturer‐specified protocols available at the time of writing and differences between helical and axial scan modes. Imaging performance was quantitatively evaluated in terms of CT number accuracy, uniformity, spatial resolution, noise, NPS, and contrast‐to‐noise ratio (CNR) in simulated soft‐tissue structures. Key results for the quantitative evaluation are visually demonstrated in both the ACR accreditation phantom (Model 464, Sun Nuclear, Melbourne, FL) and anthropomorphic phantom to relate observed trends to pertinent clinical imaging tasks. Finally, the suitability of the Airo for image‐guided brachytherapy was investigated using custom phantoms presenting a pair of applicators commonly used in cervical and endometrial cancers (interstitial ring and tandem and vaginal cylinder, respectively) in relation to surrounding simulated bone and soft‐tissue structures.

## 2 METHODS AND MATERIALS

### 2.1 The Airo system and technique protocols

**Figure 1:**
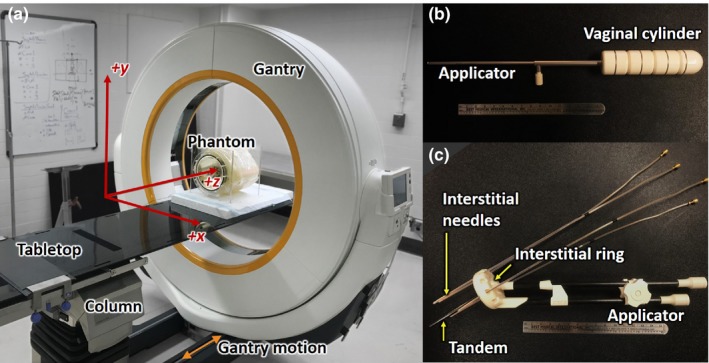
Experimental setup. (a) Mobile CT scanner (Brainlab Airo) with custom brachytherapy phantom. (b) Vaginal cylinder. (c) Interstitial ring and tandem.

The Airo is a mobile CT scanner for use in a variety of interventional settings, including spine surgery, external beam radiation therapy, and brachytherapy. The inner bore (107‐cm diameter) accommodates large body habitus and provides a fairly large working area for the surgeon or auxiliary devices about the patient — for example, stirrups employed for lithotomy positioning. The system used in this study (Model MobiCT™‐32, Software Version 3.1) was integrated with a TruSystem^TM^ 7500 surgical table (Trumpf Medical, Saalfeld, Germany) to which the tabletop locks atop an adjustable‐height column, which in turn is permanently affixed to the gantry rails. In both axial and helical scan modes, the tabletop is stationary, and the CT gantry moves along rails as illustrated in [Fig. 1(a)].

Manufacturer‐specified techniques are provided for the following body sites: head, shoulder, thorax, abdomen, pelvis, spine, upper extremities, and lower extremities. For each of these, x‐ray tube potential is fixed at 120 kV, and although 80 kV and 100 kV protocols are accessible via the nonclinical/engineering interface, the current deployment only supported air calibration for 120 kV. Rather than common protocol variations for “small/ large” body habitus or “adult/ pediatric” subjects, the x‐ray tube current automatically scales according to the weight (kg) of the patient (entered manually via the control interface).

The scanner features a detector with *N*_row_ = 32 rows, with each detector element of size *d_x_* = 0.5 mm in the lateral direction and *d_z_* = 1.06 mm in the longitudinal direction (each at isocenter). The beam width is therefore *N*_row_ × *d_z_* = 33.92 mm at isocenter. Gantry rotation time is 1.92 s. In axial scan mode, the gantry moves incrementally in Δ*z* = 31.92 mm steps/rotation (giving 1 mm overlap at each longitudinal edge of the beam). In helical scan mode, the gantry moves Δ*z* = 48 mm/rotation, giving a fixed helical pitch, *P* = (48/33.92) = 1.415. The heat capacity of 2.3 MHU permits a 50–cm‐length helical scan at maximum tube current (250 mA). The scan mAs (or effective mAs in helical mode) is given by the product of tube current (mA) and rotation time (1.92 s), divided by pitch for helical mode. For the manufacturer‐specified protocols, the x‐ray tube current in helical mode is typically increased by a factor equal to the pitch, so helical mode mAs_eff_ is equal to axial mode mAs for manufacturer‐specified technique protocols.

Image reconstruction is based on filtered backprojection (FBP) with adjustable filters referred to as “soft,” “standard,” and “sharp.” At the time of writing, the system is implemented such that the filter must be specified prior to performing the scan (and cannot be adjusted retrospectively). Transverse FOV can be selected in the range 25.6–51.2 cm. The voxel size in 3D image reconstructions ranges accordingly from *a_x_* = *a_y_* = 0.5–1.0 mm in the transverse plane (depending on the FOV — i.e., *a_x_* = *a_y_* = FOV/512) with slice thickness fixed at *a_z_* = 1.0 mm.

For consistent terminology below, the term “axial” is used in reference to axial scan mode (cf., helical scan mode), and the term “transverse” is used in reference to an (*x*, *y*) slice of the image reconstruction (cf., sagittal (*y*, *z*) or coronal (*x*, *z*) slices).

### 2.2 Dose measurements

Following the protocol described in AAPM Task Group 23 Report No. 96,^14^ dose measurements were acquired with a 100‐mm‐length (3 cm^3^) pencil ionization chamber and a Radcal electrometer (AccuDose, Radcal Corp., Monrovia, CA) with accredited calibration. Measurements were performed at the center and four cardinal peripheral locations in 16‐cm‐diameter (“head”) and 32‐cm‐diameter (“body”) acrylic cylindrical CTDI phantoms. Dose measurements were performed for the manufacturer‐specified head and pelvis scan protocols in axial mode using the 16‐cm and 32‐cm phantoms, respectively: 120 kV, 326 mAs for the head protocol, and 140 mAs for the pelvis protocol. Air kerma (mGy) at the center of each phantom (*D_c_*) and periphery of the phantom (*D_p_*, averaged over the four periphery measurements) were combined in the weighted CTDI (CTDI*_w_*) as:

(1) $${\text{CTDI}}_{w}=\left(\frac{1}{3}\right)D_{c}+\left(\frac{2}{3}\right)D_{p}$$

The volume CTDI (CTDI_vol_) was given by:

(2) $${\text{CTDI}}_{\text{vol}}=\left(\frac{1}{\text{pitch}}\right){\text{CTDI}}_{w},$$

where pitch is 1.4 for helical mode.

### 2.3 Imaging performance

Imaging performance was assessed in “Service” mode, allowing full control over the tube current (5–250 mA) and beam energy (80, 100, or 120 kV). The phantoms detailed below were used to measure image uniformity, noise, spatial resolution, etc., and each was marked with small plastic beads or tape to facilitate repositioning — for example, to repeat scans with different reconstruction filters.

#### 2.3.1 CT number accuracy and linearity

To evaluate the HU accuracy of the system, a 33‐cm diameter cylindrical Solid Water phantom (Model 467, Gammex, Madison, WI) with 16 inserts of varying electron density was scanned at 120 kV, 211 mAs in axial mode (149 mAs_eff_ in helical mode), and 25.6 cm reconstruction FOV (*a_x_* = *a_y_* = 0.5 mm; *a_z_* = 1.0 mm). Scans were acquired using the same phantom in helical mode with the standard filter and in axial mode with the soft, standard, and sharp filters. HU accuracy and linearity were assessed by comparing the measured mean HU value within each insert to the (Gammex) manufacturer‐specified HU values.

#### 2.3.2 Uniformity

Image uniformity was assessed in a 20‐cm‐diameter cylindrical water phantom scanned with a nominal technique of 120 kV, 211 mAs axial mode (149 mAs_eff_ helical mode) using soft, standard, and sharp reconstruction filters. The phantom was scanned centered at isocenter (with 25.6 cm reconstruction FOV; *a_x_* = *a_y_* = 0.5 mm; *a_z_* = 1.0 mm) and offset laterally by 5 cm (with 30 cm reconstruction FOV; *a_x_* = *a_y_* = 0.56 mm; *a_z_* = 1.0 mm). Scans were also acquired at 80 kV and 100 kV, recognizing that the system only allowed air calibration at 120 kV at the time of writing. Nonuniformity (*t*_cup_) was evaluated as the difference in average CT number measured at the center and periphery of the phantom. In the offset scan acquisition, nonuniformity was evaluated as the difference in average CT number measured at the anterior (near isocenter) and posterior periphery of the phantom.

#### 2.3.3 Spatial resolution

Spatial resolution was assessed using an edge‐spread phantom (2.8‐cm‐diameter acrylic rod in air) scanned in both helical and axial modes at 120 kV, 211 mAs axial (149 mAs_eff_ helical) with a 25.6‐cm FOV (*a_x_* = *a_y_* = 0.5 mm; *a_z_* = 1.0 mm) and reconstructed with soft, standard, and sharp filters. The oversampled edge‐spread function, ESF, was computed from images of the edge, and the numerical derivative of the ESF was computed (yielding the oversampled line‐spread function, LSF) and normalized to unity area, from which the MTF was computed by Fourier transform.^15^, ^16^

The spatial resolution of the system was also assessed qualitatively using Module 4 of the CT 464 ACR phantom (Sun Nuclear, Melbourne FL). Scans were acquired in helical mode at 120 kV, 149 mAs_eff_ and reconstructed with 25.6‐cm FOV and a sharp filter. For reference, scans were also acquired on the Philips Big Bore CT scanner (Philips, Amsterdam, Netherlands) with the following parameters: 120 kV, 149 mAs, 1.43 pitch, 16 × 0.75 collimation, 1 s rotation time, and reconstructed with 25.6‐cm FOV and a sharp filter.

#### 2.3.4 Noise and noise‐power spectrum

Noise measurements were performed using scans of the 20‐cm‐diameter water phantom (Sec. II.C.ii) acquired at 120 kV, with tube current varying from 10 mAs to 480 mAs (axial mode) and reconstructed with 25.6‐cm FOV (*a_x_* = *a_y_* = 0.5 mm; *a_z_* = 1.0 mm) with soft, standard, and sharp filters. Scans were also acquired in helical mode (149 mAs_eff_) using the same technique factors.

Noise was calculated from the standard deviation in CT number for five (5 × 5 × 5) voxel regions of interest (ROIs) sampled at fixed distance 5 cm from center. The spatial distribution in image noise (“noise maps”) was also analyzed from the standard deviation computed in ROIs (each 11 × 11 × 11 voxels) throughout the volume of reconstruction (120 kV, 83 mAs axial, (83 mAs_eff_ helical), 25.6‐cm FOV). Finally, the NPS was calculated on scans acquired at 120 kV, 144 mAs axial, (149 mAs_eff_ helical), and 25.6‐cm FOV, according to^17^:

(3) $$\text{NPS}\left(f\right)=\frac{a_{x}a_{y}a_{z}}{N_{x}N_{y}N_{z}}〈|F\left[d\left(x,y,z\right)\right]{|}^{2}〉$$

where $N_{x,y,z}$ are the size of each ROI (65 × 65 × 65 voxels), < > denotes the ensemble average of 48 ROIs, and F is the 3D discrete Fourier transform. The ROIs were taken at fixed distance 4.0 cm from center and detrended by a first‐order hyperplane to yield zero‐mean data, $d\left(x,y,z\right)$.

Scans were also acquired on the Philips Big Bore CT simulator (same parameters as Sec. II.C.iii, reconstructed with a standard filter) of the same 20‐cm‐diameter water phantom, and NPS was evaluated as described above. For comparison, a helical scan was acquired using the Airo system at 120 kV, 67.8 mAs_eff_, and reconstructed with 25.6‐cm FOV and a standard filter. The dose values (CTDI_vol_) for the Philips and Airo scans were 9.9 mGy and 10.1 mGy, respectively.

#### 2.3.5 Low contrast resolution

The low contrast resolution was assessed using a custom 16‐cm‐diameter cylindrical polyethylene phantom containing 2.8‐cm‐diameter electron density inserts (Model 467, Gammex RMI, Madison, WI) to simulate soft tissues. Scans were performed at 120 kV in axial mode with mAs varying from 10 mAs to 480 mAs with 25.6‐cm FOV. The contrast‐to‐noise ratio (CNR) was analyzed in the simulated adipose insert (AP6, $\rho_{e}^{w}$ = 0.93) relative to polyethylene background. The CNR was calculated as^18^:

(4) $$\text{CNR}=\frac{|\mu_{\text{adipose}}-\mu_{\text{background}}|}{\sigma },\;\text{where}\;\sigma =\sqrt{\frac{1}{2}\left(\sigma_{\text{adipose}}^{2}+\sigma_{\text{background}}^{2}\right)},$$

The low contrast resolution was also assessed qualitatively using Module 2 of the CT 464 ACR phantom (Sun Nuclear, Melbourne, FL). Scans were acquired at 120 kV, 149 mAs axial, (149 mAs_eff_ helical), and reconstructed with a 25.6‐cm FOV and a soft filter.

#### 2.3.6 Image quality and artifacts in anthropomorphic phantoms

To supplement the quantitative analysis, a selection of anthropomorphic phantoms was used to illustrate qualitative characteristics of imaging performance with respect to realistic anatomy. To illustrate the degree of image uniformity relative to realistic anatomical structures (including nonuniformities arising from stitching/sampling effects in helical and axial scan modes), a custom anthropomorphic body phantom was scanned using the manufacturer‐specified abdomen/pelvis technique [120 kV, 150 mAs axial mode (150 mAs_eff_ helical mode), 38.4 cm reconstruction FOV; *a_x_* = *a_y_* = 0.75 mm; *a_z_* = 1.0 mm] with standard and soft reconstruction filters. The phantom includes a natural human skeleton embedded in tissue‐equivalent Rando^TM^ plastic (The Phantom Laboratory, Greenwich NY), containing custom structures emulating the liver (with low‐contrast hepatic lesions) and pelvic region (with plastic transperineal interstitial needles).

The metal artifact reduction (MAR) algorithm available on the Airo at the time of writing was also qualitatively assessed by scanning the body phantom described above in a region of the lumbar spine containing steel pedicle screws (DePuy‐Synthes, Raynham, MA). The phantom was scanned in helical and axial scan modes using the manufacturer‐provided pelvis protocol (120 kV, 150 mAs axial, 150 mAs_eff_ helical) with a 38.4‐cm FOV and sharp reconstruction filter.

### 2.4 Brachytherapy applications

To assess performance in application to brachytherapy, two custom phantoms were constructed incorporating commonly used gynecological brachytherapy devices (Fig. 1). The first was a stainless steel (channel) and plastic (segments) vaginal cylinder (Nucletron, Veenendaal, The Netherlands) used to deliver intracavitary high dose‐rate (HDR) brachytherapy along the wall of the vaginal canal^19^ as shown in [Fig. 1(b)]. Relevant imaging tasks in placement of the vaginal cylinder include the ability to assess the applicator contact to the surrounding soft tissues and to identify the presence of air pockets.^19^ The second was an MRI/CT‐compatible interstitial ring and tandem composed of plastic (polyphenylsulfone, PPSU) (Nucletron, Veenendaal, The Netherlands) used to deliver hybrid intracavitary‐interstitial HDR brachytherapy to the cervix^20^ as shown in [Fig. 1(c)]. Relevant imaging tasks for ring and tandem brachytherapy include the ability: (a) to identify landmarks required for accurate applicator reconstruction of the tandem and ring, (b) to identify the first dwell position and source path for interstitial needles using common CT opaque brachytherapy markers, and (c) to discriminate the surrounding soft‐tissues.

Each brachytherapy device was incorporated (separately) within the 20‐cm‐diameter water phantom expanded to ~30 cm with SuperFlab (Mick Radio Nuclear Instruments, Mt. Vernon, NY) with two 4‐cm‐diameter Delrin rods placed laterally (emulating strong attenuation approximating the femoral heads). Each device was inserted in proximity to a selection of tissue‐equivalent inserts (Liver, Adipose, B‐200 Bone, Brain, Inner Bone; Gammex, Madison WI) to evaluate the effect on visibility of surrounding soft‐tissues. Scans were acquired using the manufacturer‐specified heavy pelvis protocol (120 kV, 326 mAs axial mode, 38.4‐cm FOV). Images were assessed by a brachytherapy specialized radiation oncologist and medical physicist regarding image quality with respect to pertinent clinical tasks.

## 3 RESULTS

### 3.1 Dose

Table 1 summarizes technique factors and dosimetry for various protocols. The axial mode CTDI_vol_ for the head (16 cm) and body (32 cm) protocols was 78.9 mGy and 24.9 mGy, respectively, consistent (within ~5–10%) with measurements reported by Oliver, et al.^2^ The CTDI_vol_ reported on the console based on manufacturer specifications was systematically ~20% lower than the measured values — 64.7 mGy and 20.9 mGy, respectively. The CTDI_vol_ for helical mode is scaled down from the dose in axial mode by the pitch — 56.4 mGy and 17.8 mGy for the head and body, respectively.

**Table 1:** Summary of dose measurements.

Mode	Diameter (cm)	Voltage (kV)	Current (mA)	Scan mAs	Effective mAs	D_c_ (mGy)	D_p_ (mGy)	CTDI_w_ (mGy)	CTDI_vol_ (mGy)
Axial	16	120	169.6	325.6	–	68.6	84.1	78.9	78.9
	32	120	73.0	140.2	–	10.4	32.4	24.9	24.9
Helical	16	120	–	325.6	232.6	–	–	–	56.4
	32	120	–	140.2	100.1	–	–	–	17.8

### 3.2 Accuracy and uniformity

**Figure 2:**
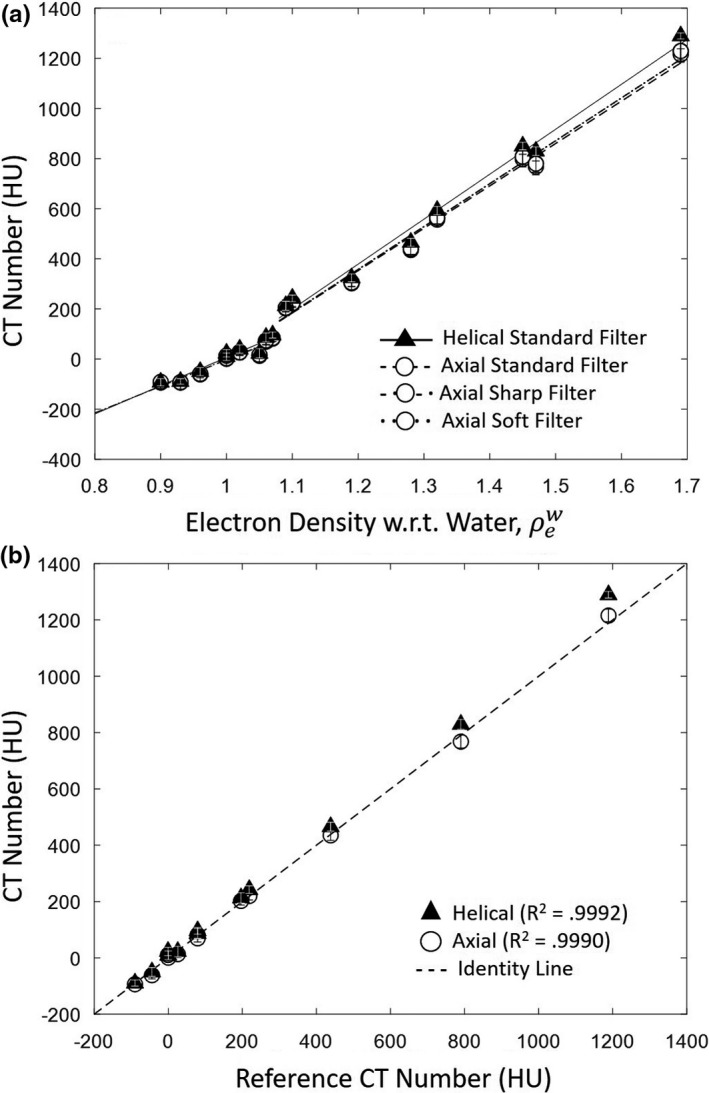
CT number linearity and accuracy. (a) Measured CT number for tissue‐equivalent inserts of varying relative electron density. Linear fits are superimposed: solid (helical, standard filter); variations of dashed and dotted (axial, all filters). (b) Measured CT number for tissue‐equivalent inserts of varying relative electron density vs reference HU values on a diagnostic CT scanner (CT/i, GE Healthcare).

The CT number linearity followed a bilinear trend with electron density as shown in [Fig. 2(a)],^21^ and CT number accuracy was unaffected by choice of filter or mAs. An increase in mean HU value was observed in helical mode, reflected in the slopes in [Fig. 2(a)] and a slight increase in mean value for higher density materials in [Fig. 2(b)]. Compared to values reported by a CT/i diagnostic CT scanner (GE Healthcare, Milwaukee, WI) shown in [Fig. 2(b)], the HU values reported by the Airo appear accurate, following the identity line with R‐squared value of 0.999 for both helical and axial modes.

**Figure 3:**
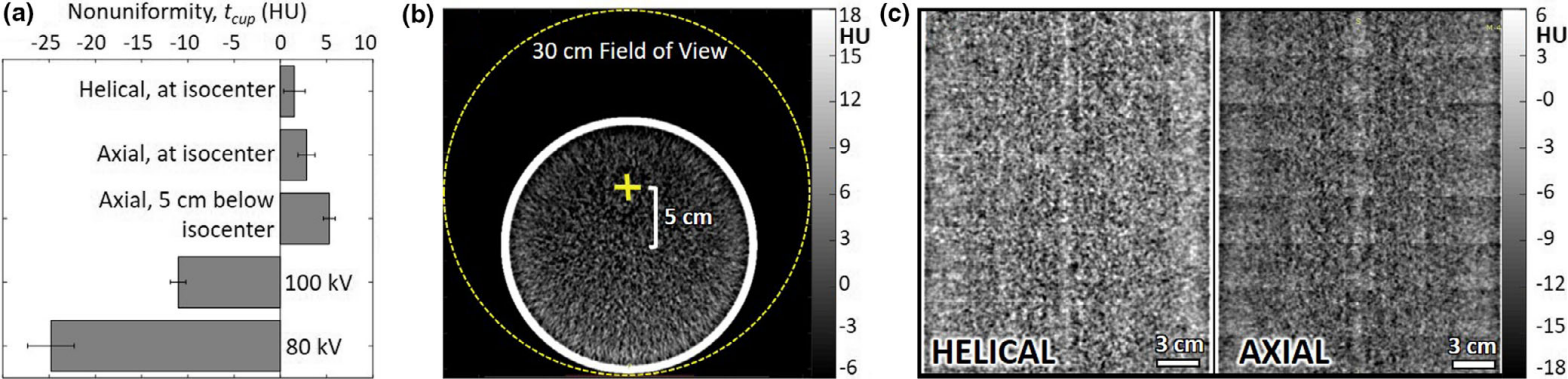
Image uniformity. (a) Magnitude of cupping in the transverse plane for helical and axial mode, averaged over three reconstruction filters. (b) Transverse slice of a 20–cm‐diameter water phantom positioned 5 cm below isocenter, showing nonuniformity introduced by positioning off isocenter. (c) Coronal slice of the water phantom, helical and axial mode, illustrating stitching artifacts for the latter.

Image uniformity measured in the 20‐cm‐diameter water phantom was not dependent on tube current or reconstruction filter. Figure 3 summarizes numerous aspects of image uniformity. As shown in [Fig. 3(a)], helical and axial modes demonstrated comparable cupping effect. A 5 cm shift of the phantom from isocenter introduced shading nonuniformity of ~ 7HU, attributed to misalignment with the bowtie filter,^22^ evident as nonuniformity in the transverse slice shown in [Fig. 3(b)]. Also shown in [Fig. 3(a)] is that the system did not support air calibration at kV settings other than 120 kV at the time of the study, and scans acquired at 80 kV or 100 kV showed correspondingly strong nonuniformity.

The effects of axial and helical scan modes on coronal and sagittal nonuniformity were evident in the water phantom [Fig. 3(c)]. Figure 3c shows the slightly higher mean HU value observed in helical mode (shown also in Fig. 2) as well as a stitching artifact in axial scan mode. Such stitching was largely absent in helical mode, of course.

### 3.3 Spatial resolution — MTF

**Figure 4:**
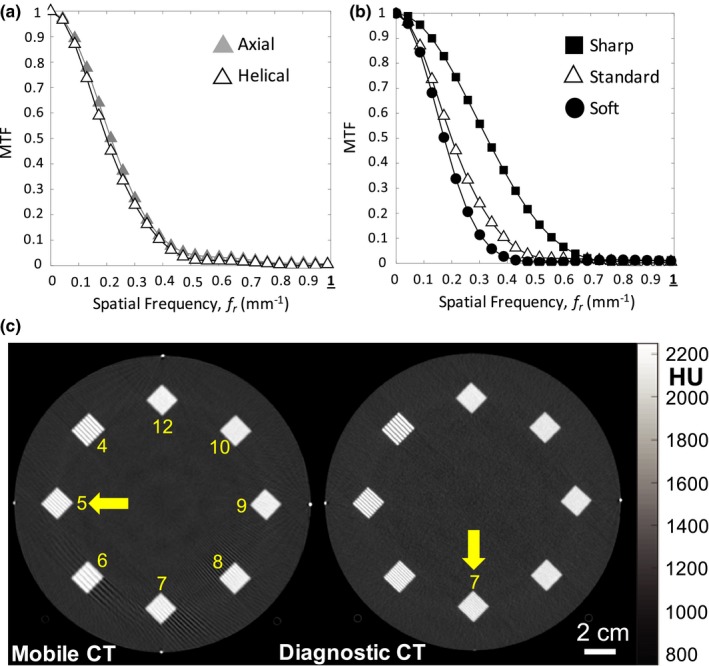
Spatial resolution. (a) MTF for helical and axial mode (standard filter). (b) MTF for helical scan mode for various reconstruction filters. (c) Images of line‐pair patterns in the ACR accreditation phantom for helical scan protocols from the Airo (left) and Philips Big Bore (right). MTF, modulation transfer function.

Axial mode and helical mode demonstrated comparable spatial resolution, as shown in [Fig. 4(a)] for the standard reconstruction filter. In each case, MTF is reduced to 10% at ~0.38 mm^−1^. A rough estimate of limiting resolution is therefore ~(1/2/0.38) = 1.3 mm feature size. The effect of reconstruction filter is shown in [Fig. 4(b)], with the sharp reconstruction filter giving highest MTF, falling to 10% at ~0.55 mm^−1^ and limiting resolution down to ~(1/2/0.55) = 0.9 mm feature size, consistent with previously published work.^1^, ^2^

The spatial resolution was also visualized in images of the ACR phantom line‐pair patterns as shown in [Fig. 4(c)]. The Airo mobile CT scanner demonstrated resolution up to the ~5 lp/cm group, whereas the Philips Big Bore CT scanner demonstrated visualization up to the ~7 lp/cm group. Streak artifacts evident in the mobile CT scan image are further investigated via the anthropomorphic phantoms discussed below (and in Fig. 9).

### 3.4 Noise

**Figure 5:**
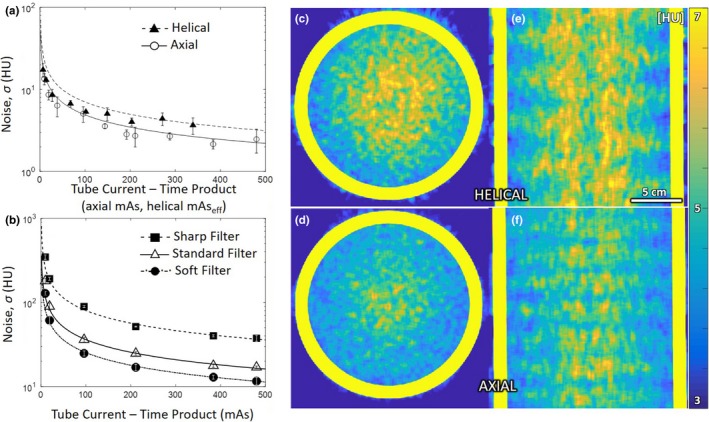
Image noise. (a) Noise measured as a function of mAs (or mAs_eff_) in a 20‐cm‐diameter water phantom. (b) Noise measured for various reconstruction filters for axial mode. The spatial distribution “map” of image noise is shown in transverse and coronal planes in (c,e) helical and (d,f) axial modes (each for the standard filter).

As shown in [Figs. 5(a)5(b)], image noise followed an expected inverse square‐root relationship with dose. Even accounting for pitch (i.e., comparing axial mAs with helical mAs_eff_), helical scan mode exhibited slightly increased noise (~2 HU) compared to axial scan mode. The effect may be attributed to slight differences in calibration or reconstruction, evident also in increased mean HU value in Fig. 2. The noise varied with reconstruction filter as shown in [Fig. 5(b)].

The spatial distribution of image noise is shown in [Figs. 5(c)5(f)]. Each shows a slight elevation in noise at the center of the phantom (consistent with increased attenuation and suggesting conservative matching by the bowtie filter^22^). The increased noise for helical scan mode is also evident. Each mode also exhibits a horizontal banding in the noise magnitude evident in coronal planes [Figs. 5(c)5(f)]. These effects may result from imperfect detector air calibration at the longitudinal edges of the detector adjacent to the collimator [evident as stitching artifacts in Fig. 3(d)].

**Figure 6:**
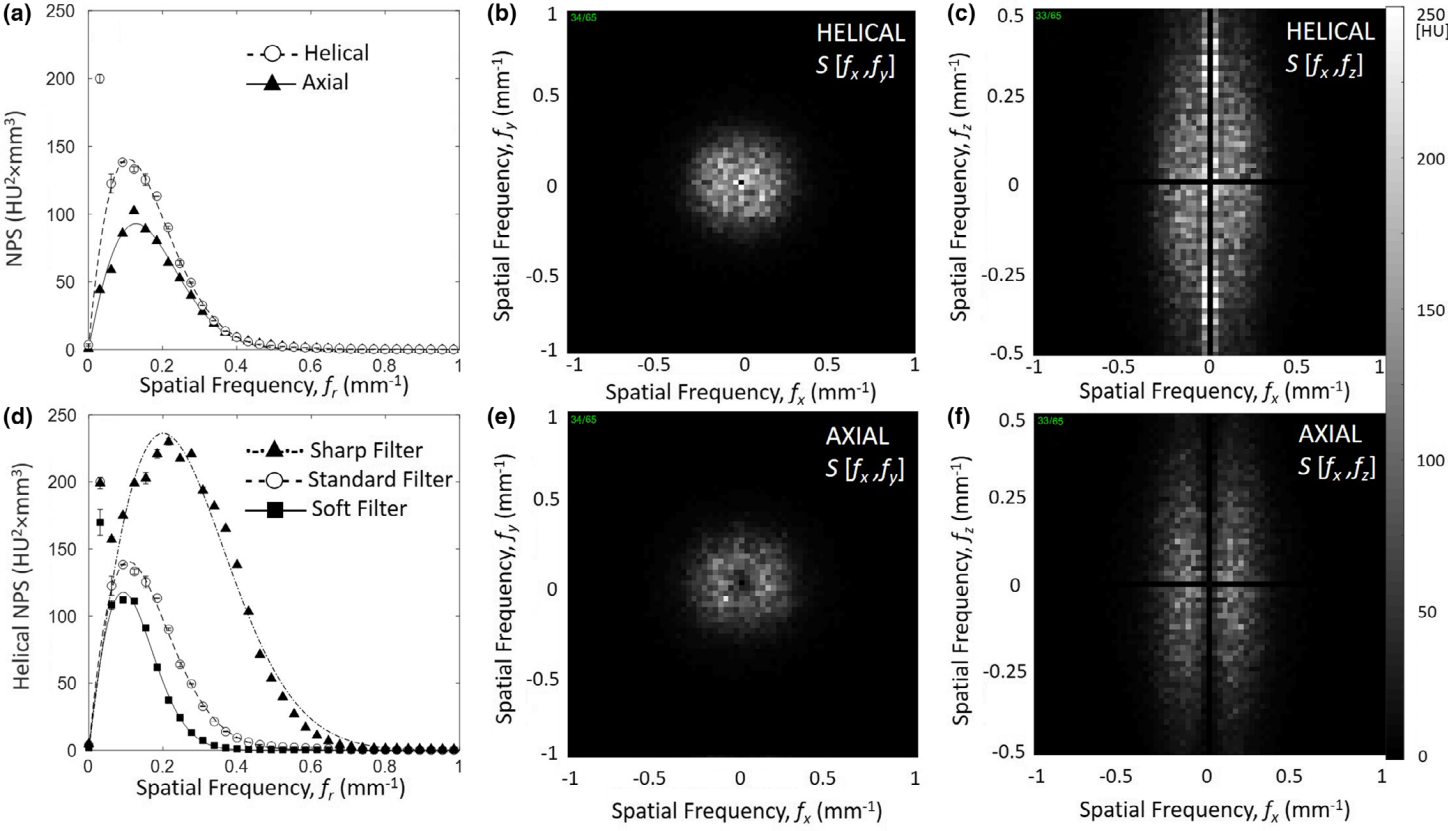
NPS for various scan modes and reconstruction filter. (a) Transverse plane NPS in helical and axial modes with standard reconstruction filter. (b) Transverse plane NPS for helical mode and three reconstruction filters. (c) Transverse (*f_x_*,*f_y_*) and coronal (*f_x_*,*f_z_*) plane NPS are shown in (c‐f) for helical and axial modes (each with standard reconstruction filter). NPS, noise power spectrum.

As shown in Fig. 6, the NPS for helical mode is greater than that in axial mode for similar mAs/mAs_eff_, exhibiting a slightly increased low‐ to mid‐frequency noise characteristic [Fig. 6(a)]. Helical scans also presented a challenge to ROI detrending, evident by the outliers at low frequency in [Figs. 6(a)6(b)] and near the *f_x_* axis in [Figs. 6(c),6(e)]. The increased low‐frequency noise for helical mode is attributed to the nonuniformities evident in Fig. 5.

NPS varied as expected with reconstruction filter as shown in [Fig. 6(b)], where the sharp filter exhibited an increase in mid‐ to high‐frequency noise, and conversely, the soft filter reduced mid‐ to high‐frequency noise.

**Figure 7:**
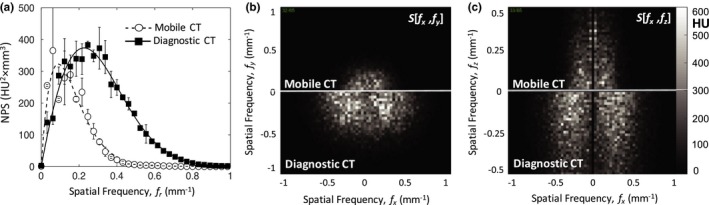
Image NPS for the mobile CT (Airo) in comparison to diagnostic CT (Philips Big Bore). (a) Transverse plane NPS in helical mode, each with “standard” reconstruction filter and comparable dose (10.1 and 9.9 mGy, respectively), illustrating a factor of ~2 in spatial‐frequency cutoff between the two systems. (b) Transverse (*f_x_*,*f_y_*) and (c) coronal (*f_x_*,*f_z_*) plane NPS for the Airo (top) and Big Bore CT (bottom), each with “standard” reconstruction filter and comparable dose. NPS, noise power spectrum.

Figure 7 shows a comparison of the NPS measured for the mobile CT scanner (Airo) and an established diagnostic‐quality CT scanner (Philips CT Big Bore). At comparable dose, the NPS for the helical mobile CT scan was less than that of the diagnostic CT scan, exhibiting a decrease in mid‐ to high‐frequency noise characteristics [Fig. 7(a)]. The reduced NPS characteristic for the mobile scanner is consistent with reduced spatial resolution (spatial‐frequency cutoff ~0.4 mm^−1^ for the Airo compared to ~0.8 mm^−1^ for the Big Bore CT). The helical mobile CT scan demonstrated increased low‐frequency noise, attributable to the nonuniformities in Fig. 5. The reduced mid‐ to high‐frequency noise and increased low‐frequency noise in mobile CT scans are also evident in the transverse and coronal plane depictions of the NPS [Fig. 7(b,c)].

### 3.5 Low contrast resolution

**Figure 8:**
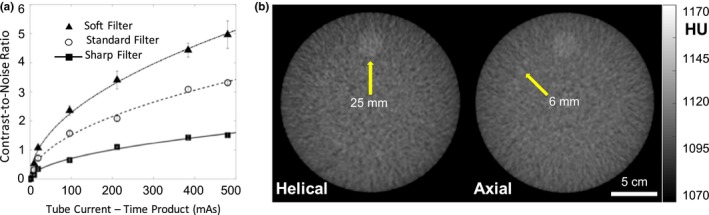
Low‐contrast performance: CNR and simulated soft‐tissue visualization. (a) CNR between simulated adipose and polypropylene background measured in a 16‐cm phantom. (b) Example helical and axial mode images of low contrast resolution inserts (Module 2) in ACR 464 CT accreditation phantom. CNR, contrast‐to‐noise ratio.

As shown in [Fig. 8(a)], the CNR followed the expected square‐root relationship with dose, consistent with Fig. 5, and contrast (i.e., mean difference in HU) was independent of dose. The CNR also improved as expected with smoother reconstruction filters. Figure 8b illustrates the detectability of a low‐contrast insert (6 HU contrast to background) for the ~25 mm diameter cylinder in helical mode, compared to the ~6 mm diameter cylinder in axial mode, consistent with the increased noise levels observed in helical mode (as in Figs. 5, 6).

### 3.6 Image quality and artifacts in anthropomorphic phantoms

**Figure 9:**
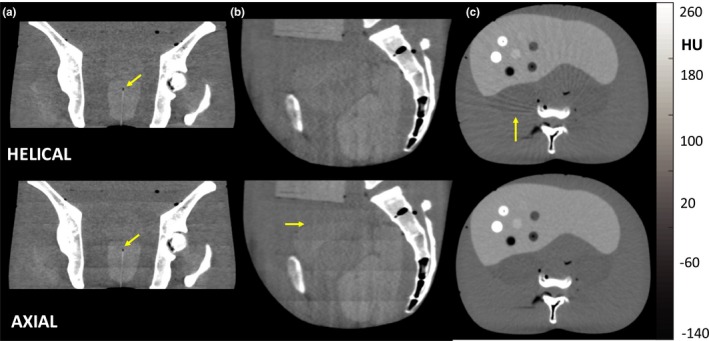
Soft‐tissue visualization and artifacts in an anthropomorphic phantom. Example helical and axial mode images of (a) coronal slices in regions of the pelvis, including an interstitial brachytherapy catheter (standard filter), (b) sagittal slices in pelvic region (soft filter), and (c) transverse slices of an abdomen phantom in regions containing a variety of low‐ and high‐contrast spheres in the liver (soft filter). Note the windmill artifacts evident about high‐contrast, high‐frequency structures such as the vertebrae for helical mode (c) and the stitching artifacts evident in coronal and sagittal planes for axial mode (a, b).

The artifacts and image characteristics described in Figs. 2, 3, 4, 5, 6, 7, 8 are illustrated in anthropomorphic phantoms representing clinically relevant anatomy. As shown in Fig. 9, the influence of stitching artifacts described in Section III.B. is seen in [Fig. 9(a)9(b)], where such artifacts could challenge accurate delineation of the superior/inferior aspect of anatomical structures. The effect did not impair identification of interstitial brachytherapy catheters in the prostate, evident in the coronal views of [Fig. 9(a)], and helical and axial scan modes performed comparably for this task.

The low‐contrast, soft‐tissue capability was further illustrated in transverse body phantom images in [Fig. 9(c)], where helical mode exhibited windmill artifacts (evident as streaks arising from high‐frequency bone structures) associated with the fairly high level of pitch.^23^ The ability to visualize the (spherical) liver nodules in the transverse view was comparable between helical and axial scan modes.

**Figure 10:**
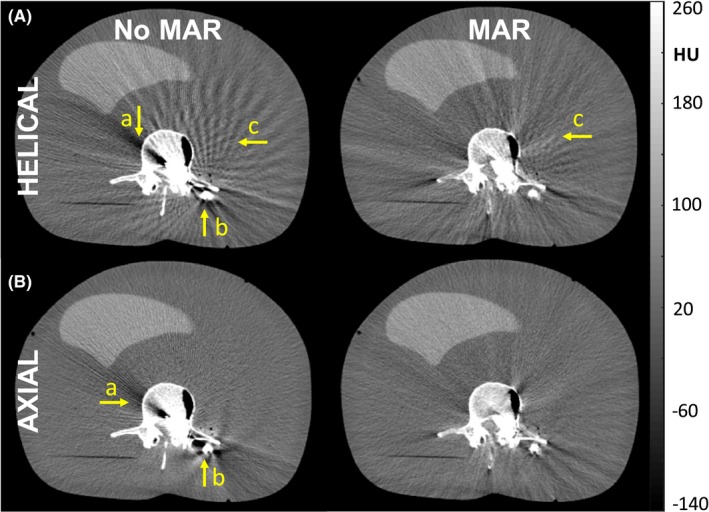
Images of an anthropomorphic phantom containing surgical instruction. (A) Helical mode: although the MAR algorithm reduces shading and streak artifacts associated with beam hardening (arrows (a) and (b)), helical mode is susceptible to windmill artifacts about high‐contrast, high‐frequency structures (i.e., surgical instrumentation) that are not addressed by MAR. (B) Axial mode: the MAR algorithm is seen to reduce shading and streaks associated with beam hardening (arrows (a) and (b)). MAR, metal artifact reduction.

Figure 10 illustrates the performance of the MAR algorithm available on the system at the time of deployment, illustrating the reduction in streak artifact for both helical and axial modes. For helical mode, streaks arise from the high‐frequency structure associated with the metal screw [Fig. 10(A‐a,b)] due to a combination of beam‐hardening, photon starvation, and helical z‐interpolation/sampling effects.^22^, ^23^, ^24^ The MAR algorithm treats the former (beam‐hardening and photon starvation) but not the latter, so the helical mode image exhibits streaks associated with windmill artifacts [Fig. 10(A‐c)] even with MAR. In axial mode, streaks arise primarily from beam‐hardening and photon starvation,^22^, ^24^ so MAR better addresses the conspicuous artifact [Fig. 10(B‐a,b)]. The ability to delineate the boundaries of the screw (e.g., identify pedicle breach) was still challenging even with MAR, especially in helical mode where windmill artifacts arising from the screw presented strong nonuniformity.

### 3.7 Brachytherapy Applications

**Figure 11:**
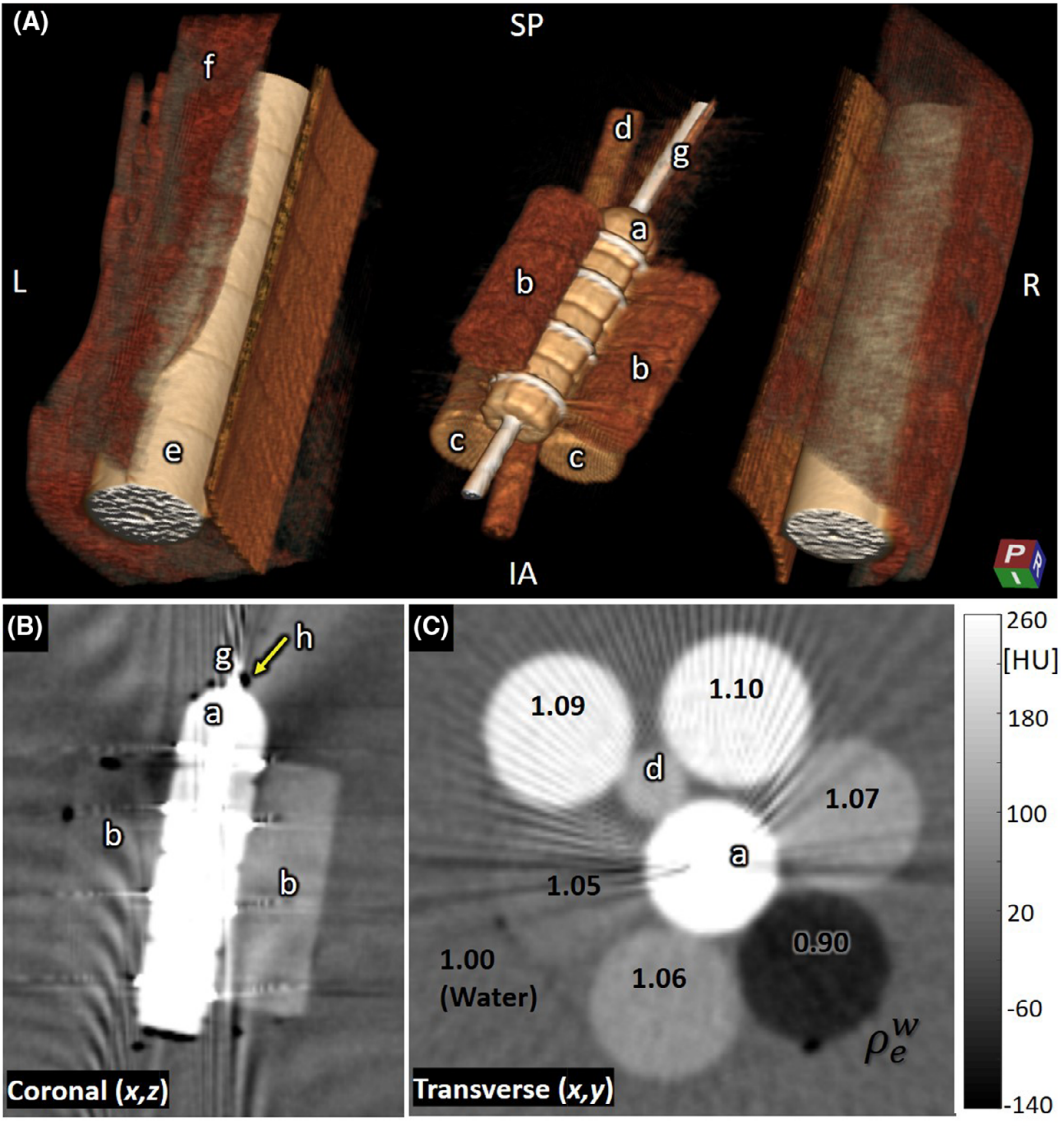
Brachytherapy phantom: vaginal cylinder. (A) 3D rendering of (a) vaginal cylinder, (b) low‐density tissue‐simulating inserts, (c) higher density bone‐simulating inserts, (d) plastic support rod, (e) Delrin rods (simulating femurs), (f) Superflab, and (g) stainless steel vaginal tube. (B) Coronal slice showing the vaginal cylinder and surrounding low‐density inserts as well as an air pocket (h) visible at the tip of the cylinder. (C) Transverse slice showing the vaginal cylinder and surrounding tissue‐simulating inserts (each labeled according to relative electron density).

**Figure 12:**
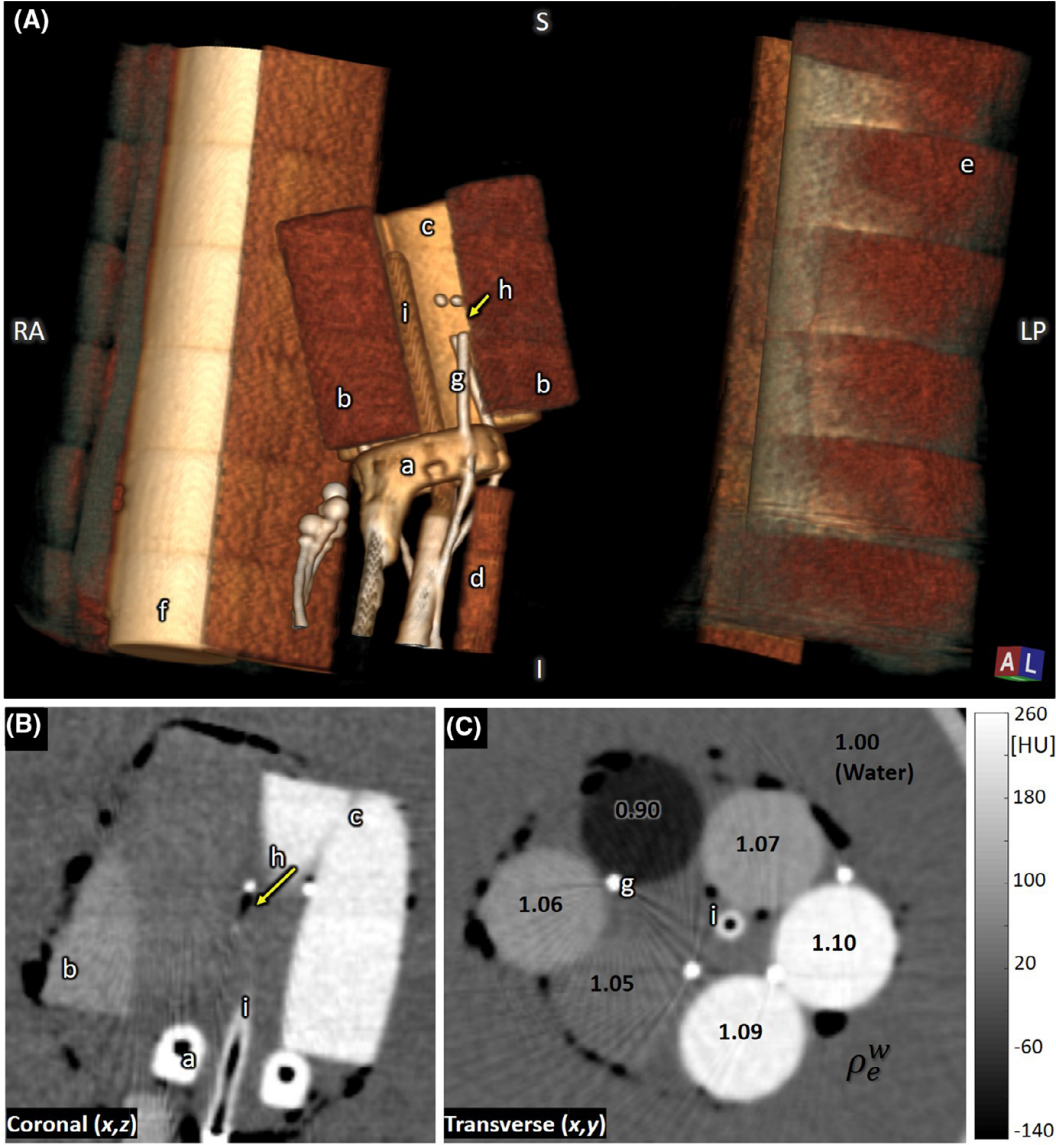
Brachytherapy phantom: interstitial ring and tandem. (A) 3D rendering of (a) interstitial ring, (b) low‐density tissue‐simulating inserts, (c) higher density bone‐simulating insert, (d) plastic support rod, (e) Superflab, (f) Delrin rods (simulating femurs), (g) interstitial needles, (h) first dwell position marker, and (i) tandem rod. (B) Coronal slice showing the interstitial ring, low‐density inserts, high‐density inserts, first dwell position, and tandem rod. (C) Transverse slice showing interstitial needles, tandem rod, and tissue‐simulating inserts (each labeled according to relative electron density).

As illustrated in Figs. 11, 12, images of custom brachytherapy phantoms demonstrate image quality suitable to a number of pertinent tasks associated with guidance of intracavitary and interstitial brachytherapy. Based on the studies described in Fig. 10 (showing only modest improvement for MAR), images were acquired without the current MAR algorithm, consistent with our deployment in translation to routine clinical use. Figure 11A depicts a 3D rendering of the vaginal cylinder surrounded by soft‐tissue‐simulating inserts. Among the pertinent clinical tasks is the ability to assess intimate contact (lack of air cavity) between the vaginal cylinder and adjacent soft tissue. Despite the fairly strong streaks from the vaginal cylinder seen in [Fig. 11(B)], an air pocket at the top of the cylinder is easily identified. Also, surrounding soft‐tissue structures are fairly well visualized despite streaks from the stainless steel tube in the cylinder [Fig. 11(C)]. The superior surface of the vaginal cylinder is well defined and can be identified for applicator reconstruction. Given the severity of windmill artifacts arising from metal instrumentation in the brachytherapy devices, examples shown below were performed in axial mode.

Figure 12A shows a 3D rendering of the interstitial ring and tandem surrounded by soft‐tissue inserts embedded within the custom brachytherapy phantom. The interstitial needles contain markers denoting radioactive source dwell positions. Among the pertinent clinical tasks is the ability to identify applicator landmarks which would permit applicator reconstruction. The lumen of the ring and tandem are clearly visible [Fig. 12(B)] for applicator reconstruction. The brachytherapy CT markers placed in the interstitial needles allow quick and accurate catheter digitization [Fig. 12(C)]. Additionally, the first dwell position landmark of the interstitial needle is clearly identifiable (arrow in [Fig. 12(B)]. Soft‐tissue structures with electron density below 0.95 or greater than 1.05 were also easily visualized [Fig. 12(C)].

## 4 DISCUSSION AND CONCLUSIONS

Technical assessment of the imaging performance and dose for new clinical imaging systems provides an important guide to their safe, effective deployment, providing a check that technique protocols are within expectations of image quality and dose and helping to identify factors that may warrant additional attention from the manufacturer, medical physicists, or radiology technicians. Especially for relatively new systems for which acceptance testing standards may not yet be well developed, technical assessment in terms of quantitative metrology is important to ensure safe, effective operation, and provide a baseline for future quality assurance tests. The Airo mobile CT scanner is such a system — particularly in application to brachytherapy — and the technical assessment reported above helps to better inform its clinical deployment.

Among the attractive characteristics of the Airo are its mobility and compact design. These attributes were important criteria in siting a system within a small HDR suite. Additionally, the large open bore of the scanner allows patients to be scanned in the dorsal lithotomy position, as it accommodates stirrups if needed. At the time of this work, a variety of limitations in functionality were evident but did not necessarily detract from the value of the system in clinical use — for example, a limited range of technique protocols (fixed 120 kV), helical pitch (fixed 1.4), and the need to select reconstruction filter prior to the scan (without opportunity to re‐reconstruct with a different filter).

Technical measures of imaging performance included uniformity, HU accuracy, linearity, spatial resolution, and noise — each characterized in simple phantoms for quantitative analysis and visualized in qualitative anthropomorphic phantoms presenting pertinent (simulated) tissues and interventional devices. In both helical and axial scan modes, the system demonstrated a high degree of CT number accuracy and linearity, which facilitates model‐based dose calculation in brachytherapy applications.^25^

Image uniformity for axial mode suffered primarily by edges and gradients in the *z* (longitudinal) direction, evident as “stitching” artifacts in sagittal or coronal planes, attributed to nonuniformity in fluence and/or detector calibration at the ±z extent of the multirow detector. Such nonuniformities were of similar magnitude to the contrast in soft tissues and could diminish visualization in sagittal/ coronal views. In helical mode, windmill artifacts associated with the fairly high pitch and arising from high‐frequency, high‐contrast structures (e.g., bones or metal implants) also diminished visualization of low‐contrast soft tissues. For phantoms containing metal implants (e.g., a pedicle screw), the manufacturer‐provided MAR algorithm was found to reduce the severity of metal streaks in axial mode, but windmill artifacts in helical mode were persistent at a level that confounded visualization of nearby tissues and markers.

Apart from such artifacts, image noise followed expected quantum noise behavior (inversely proportional to square root of dose), and simulated soft tissues (e.g., fat, muscle, liver, etc.) could be clearly delineated. Spatial resolution was consistent with visualization of high‐contrast structures down to ~0.9–1.3 mm feature size, depending on choice of reconstruction filter.

The radiation dose associated with manufacturer‐specified technique protocols was characterized — an important consideration for clinical use, particularly since the current implementation requires a rescan if the choice of reconstruction filter is to be adjusted. The axial mode body protocol yielded CTDI_w_ = CTDI_vol_ = 24.9 mGy (central and peripheral weighted air kerma in a 32‐cm‐diameter acrylic cylinder), which is consistent with the dose for AAPM reference protocols for imaging of the adult abdomen‐pelvis (15–25 mGy) and the ACR reference level of 25 mGy. The axial mode head protocol yielded CTDI_w_ = CTDI_vol_ = 78.9 mGy (central and peripheral weighted air kerma in a 16‐cm‐diameter acrylic cylinder), which is 20–40% higher than the AAPM reference protocol dose for head scanning (55–65 mGy) and is above the ACR reference level of 75 mGy (and just below the ACR limit of 80 mGy).

Clinical deployment of the scanner in brachytherapy was informed by the technical assessment, noting potential improvements and helping to guide technique selection. The anthropomorphic phantoms — although small compared to typical body habitus in gynecological brachytherapy — indicated that image quality was suitable to common brachytherapy‐related tasks — for example, judging intimate contact of a vaginal cylinder with adjacent tissue or localizing landmarks used for applicator reconstruction in treatment planning. Windmill artifacts arising from high‐frequency, high‐contrast metal implants diminished the utility of helical mode, and despite the increased scan time, axial mode acquisition was identified as the nominal technique. In addition, use of the manufacturer‐specified heavy pelvis protocol was not recommended for routine scanning, due to the modest heat capacity resulting in tube cooling times of up to ~20 min and subsequently delaying the acquisition of a second scan if needed. Lastly, the MAR algorithm was not recommended in its current form, as it interfered with the ability to reliably identify the copper markers in the interstitial needles during clinical use.

The advantages of a mobile CT scanner (e.g., compared to real‐time ultrasound or a diagnostic MDCT located in a separate imaging suite) for brachytherapy guidance were also evident from this work. Chief among these is the ability to image and treat the patient without repositioning, the small footprint of the scanner, and the feasibility of moving the scanner out of the room. Likely improvements to workflow are also anticipated (but not measured directly in the current work) by reducing time requirements in patient transfer to the brachytherapy suite. Moreover, the scanner offers the clinician the ability to verify applicator position immediately prior to treatment delivery without disturbing the treatment position.

A limitation of the current work is that the phantom measurements did not probe image quality factors related to patient motion. The studies therefore did not assess potential benefits to image quality associated with faster scan speed in helical mode. Future work includes evaluation of clinical workflow with the system and potential improvements to brachytherapy treatment outcomes. One may also anticipate future improvements to the system from the manufacturer, including lower‐dose protocols, an increased range of technique factors (e.g., selection of kV and pitch), the ability to reconstruct a dataset with any reconstruction filter, improved longitudinal image uniformity (reduced stitching artifacts), and improved MAR methods.

## CONFLICT OF INTEREST

No conflict of interest.
